# 501 A Change to Virtual Peer Supporter Training and its Effect on Participant Readiness and Satisfaction

**DOI:** 10.1093/jbcr/irac012.132

**Published:** 2022-03-23

**Authors:** Sandra J Cramolini, Jessica Irven, Pam Peterson

**Affiliations:** Phoenix Society for Burn Survivors, Knoxville, Tennessee; Phoenix Society for Burn Survivors, Durham, North Carolina; Phoenix Society for Burn Survivors, Grand Rapids, Michigan

## Abstract

**Introduction:**

Peer support gives burn survivors and their loved ones hope for a brighter future helping speed and strengthen recovery for burn survivors and their loved ones. A strategic plan to explore a digital program change from in-person increasing access for burn survivors and burn programs was scheduled for the third quarter of 2021, but the global pandemic expedited the implementation. A 2-week asynchronous and a 4-hour virtual course was designed. This study sought to evaluate the effectiveness and participants' perceptions of asynchronous and virtual training and their post-course readiness in the role of peer supporter.

**Methods:**

A 5-point Likert scale was used to evaluate the opinions, perceptions, and satisfaction of the participants. The scale, ranging from strongly agree to disagree strongly, evaluated the modular education (length, relationship to manual, preparation for the virtual course) and the usefulness of a condensed online manual. A 5-point Likert scale (ranging from extremely helpful to not at all helpful) was used to evaluate each component of the course group exercises and the participants' perception of skill development to become peer supporters. Open ended questions collected additional feedback to analyze potential changes to the new platform. The metric Net Promoter Score, or NPS, was used to measure the recommendation of the new program to others.

**Results:**

Burn survivors (n=41) and medical professionals (n=3) participated in 6 courses over eight months. Participants reported the manual helped prepare for the course (86% strongly agree and 14% agree). Respondents felt the asynchronous modular education prepared them for the virtual component of the class (75% strongly agree and 25% agree). Additionally, respondents felt the 2-week timeline for module completion met their expectations (98% yes and 2% no). The participants felt the overall length of the course was just right (89%) to 9% too short and 2% too long. Participants rated their readiness (confidence level) to utilize their learned skills to support another burn survivor (52% extremely confident, 39% very confident, and 9% confident) after the virtual class. The combined courses rated an 82 NPS, equating to a positive participant experience and a course recommendation to others.

**Conclusions:**

This study concluded the asynchronous and virtual education course provided an effective method to train burn survivors to become peer supporters. Overall, burn survivors expressed confidence in utilizing the skills learned from the new training program. Participants also experienced the use of virtual tools to meet the needs of burn survivors for peer support.

